# Principal Component Analysis Applied to Digital Pulse Shape Analysis for Isotope Discrimination

**DOI:** 10.3390/s23239418

**Published:** 2023-11-26

**Authors:** Katherine Guerrero-Morejón, José María Hinojo-Montero, Fernando Muñoz-Chavero, Juan Luis Flores-Garrido, Juan Antonio Gómez-Galán, Ramón González-Carvajal

**Affiliations:** 1 Department of Electronic Engineering, University of Sevilla, 41092 Sevilla, Spain; kguerrero@us.es (K.G.-M.); jhinojo@us.es (J.M.H.-M.); fmunoz@us.es (F.M.-C.); carvajal@us.es (R.G.-C.); 2 Department of Electrical and Thermal Engineering, University of Huelva, 21007 Huelva, Spain; juan.flores@dfaie.uhu.es; 3 Department of Electronic Engineering, Computers, and Automation, University of Huelva, 21007 Huelva, Spain

**Keywords:** support vector machine (SVM), principal component analysis (PCA), isotopes discrimination, digital pulse shape analysis (DPSA), machine learning (ML), edge computing

## Abstract

Digital pulse shape analysis (DPSA) techniques are becoming increasingly important for the study of nuclear reactions since the development of fast digitizers. These techniques allow us to obtain the (A, Z) values of the reaction products impinging on the new generation solid-state detectors. In this paper, we present a computationally efficient method to discriminate isotopes with similar energy levels, with the aim of enabling the edge-computing paradigm in future field-programmable gate-array-based acquisition systems. The discrimination of isotope pairs with analogous energy levels has been a topic of interest in the literature, leading to various solutions based on statistical features or convolutional neural networks. Leveraging a valuable dataset obtained from experiments conducted by researchers in the FAZIA Collaboration at the CIME cyclotron in GANIL laboratories, we aim to establish a comparative analysis regarding selectivity and computational efficiency, as this dataset has been employed in several prior publications. Specifically, this work presents an approach to discriminate between pairs of isotopes with similar energies, namely, ^12,13^C, ^36,40^Ar, and ^80,84^Kr, using principal component analysis (PCA) for data preprocessing. Consequently, a linear and cubic machine learning (ML) support vector machine (SVM) classification model was trained and tested, achieving a high identification capability, especially in the cubic one. These results offer improved computational efficiency compared to the previously reported methodologies.

## 1. Introduction

Technological breakthroughs in particle detectors and the development of new radioactive ion beam facilities (RIBFs), along with advances in machine learning (ML) and artificial intelligence (AI), have made particle, isotope, and ion classification techniques increasingly relevant in nuclear physics research. These techniques are crucial to discard contaminant beams [1]. Additionally, they must be computationally efficient to be executed in real time, reducing the amount of data to be transmitted, stored, and processed.

On the one hand, the construction and operation of new and upgraded RIBFs, such as FAIR [2], EURISOL [3], SPES [4], EXOTIC [5], or SPIRAL [6], will enable the study of new exotic features of the nuclear structure due to the availability of high-intensity radioactive ion beams. On the other hand, the continuous improvement in the spatial and temporal resolution of silicon detectors, which are the main semiconductor used due to the fact that the gap between the valence and conduction bands is 1.12 eV, has led to improved accuracy in particle energy measurement [7]. This has made it possible to distinguish isotopes whose energies are very close.

In addition to the improvement of these two key elements for advancing our understanding of nuclear structure, there is a constant growth in the computational capacity and speed provided by devices such as FPGAs. They can perform complex and highly demanding operations at the first level of processing where the data acquisition system (DAQ) or the event selector are located [8], enabling the edge-computing paradigm in the nuclear physics field. To achieve this purpose, efficient isotope discrimination algorithms are vital. For this purpose, different techniques have been published in the literature. Some authors have used time-of-flight (ToF) to identify and classify the nature of the isotope under study [9,10]. This technique is based on measuring the time difference between two given time stamps (either the start and stop time stamp—requiring a detector close to the source and a second detector located at a certain distance for its implementation—or the stop time stamp and the start time stamp of the accelerator radiofrequency, RF—note that this method is only valid in those facilities that implement pulsed beams) [9]. The main drawback of this technique is that it requires a complex clock distribution network to ensure that all detectors receive exactly the same clock signal, resulting in complex control and acquisition systems such as those implemented in FAZIA [11,12].

A second technique reported in the literature corresponds to the measurement of the ionization energy loss [9]. This technique is based on the relation of the momentum of the particle with its velocity, which is estimated after measuring the ionization energy losses. Nevertheless, this method presents two major challenges that make particle detection difficult and limit its application: Two particles, with different masses but the same momentum, generate the same energy loss and the saturation of the detector itself.

Finally, a third method for isotope discrimination is known as pulse shape analysis (PSA), which is a powerful technique for characterizing and distinguishing particles or isotopes based on the unique waveforms they generate after impinging on a particle or scintillation detector [13,14,15,16,17,18,19,20,21]. A very promising variant of this technique is its digital implementation, known as digital PSA [22,23,24,25,26]. It leverages the benefits of digital signal processing to achieve reliability and inclusion of advanced algorithms such as artificial neural networks (ANN), [27,28], or machine/deep learning algorithms, [29,30], making it a preferred choice for many modern applications in nuclear physics, radiation detection, and related fields. The latter technique has demonstrated high performance in classifying isotopes, even when their energy and atomic weight are very similar. An example of this performance is given in [28], where the isotope pairs ^12,13^C, ^36,40^Ar, and ^80,84^Kr were identified with a hit rate close to 91%. Furthermore, this technique, due to the high computational cost it requires, will benefit greatly from devices such as FPGAs, as they offer the possibility of running multiple operations in parallel at a low cost [27,31].

This research work proposes a new classifier for PSA based on SVM ML algorithms that improves the accuracy in the discrimination of fragments generated in nuclear reactions with similar energies, reducing the number of computational resources used. In order to perform this task, it is required to collect a large dataset for different values of the mass number (A), atomic number (Z), and energy. Specifically, the dataset used in this work was acquired from experimental measurements using the CIME cyclotron at GANIL [22,27,28]. Thus, in order to discriminate the different isotopes, the dataset is preprocessed using principal component analysis (PCA) to reduce the amount of information that the SVM algorithm must process, obtaining a solution that can be easily integrated into a physical system such as an FPGA without compromising the discrimination results. This last requirement is vital for the application of this method in array detectors containing a large number of single silicon detectors, working simultaneously to process a huge amount of data.

The rest of the document is as follows: Section 2 describes the acquisition of the dataset used to train and assess the proposed classifier. Section 3 details the proposed classifier based on the SVM algorithm, as well as the preprocessing of the dataset. Section 4 collects the results obtained by applying the proposed method. Section 5 presents a comparison of the results with other techniques published in the literature. The results are also discussed in this section. Finally, in Section 6, some conclusions are drawn and future work is outlined.

## 2. Data Source and Acquisition Description

**Figure 1:**
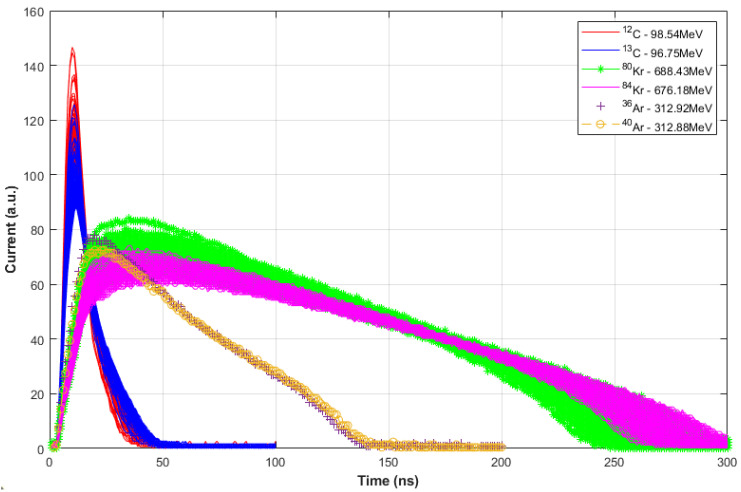
Dataset of the three pairs of isotopes: ^12,13^C, ^36,40^Ar, and ^80,84^Kr.

As described in the previous section, the dataset used in this work corresponds to the results obtained from measurements at GANIL using CIME cyclotron-accelerated ions. This experiment focuses on the electric current signal produced by the detected particle. They used a silicon neutron transmutation doped (NTD) detector of 300 μm thickness and 200 mm${}^{2}$ active area, collimated to a 10 mm diameter. In this experiment, no target was required because the detector was placed above the beam line to collect the ions directly. The detector was mounted in an inverse configuration because, according to the authors, this configuration increases the plasma time differences for ions of a given energy [14]. A voltage of 190 V was applied during the experiment, and the depletion voltage was 140 V. The detector was connected to a PACI low-gain pre-amplifier, operating with a bandwidth of 300 MHz [26], and placed very close, exactly 4 cm away, to avoid significant signal degradation, even inside the vacuum chamber. The outputs provided by this amplifier are proportional to the charge and current produced by the detected particle and were sent to a commercial 8-bit ACQIRIS digitizer [32], operating at a sampling frequency of 2 GHz. This ACQIRIS acquisition system stored all the signals from the different ions using the same amplitude scale for direct analysis and comparison. They then measured the energy with a peak sensing ADC, connecting the charge output of the PACI to standard shaping analog electronics. The beam energy in the experiment ranged from 7.39 AMeV to 8.68 AMeV and the accelerated ion species covered a somewhat wide range, from ^12^C to ^84^Kr. In each run of the experiment, they used “mixed” beams with known isotopes, all with different mass and charge-to-mass ratios but with the same energy per nucleon. The identity of each pulse and the particle mass number were determined by measuring the total energy. A more detailed description of the experiment can be found in [22]. Within their results, the authors have managed to find three pairs of isotopes with very similar total energy: ^12^C at 98.54 MeV vs. ^13^C at 96.75 MeV; ^36^Ar at 313.92 MeV vs. ^40^Ar at 312.88 MeV and ^80^Kr at 688.43 MeV vs. ^84^Kr at 676.18 MeV. Figure 1 shows the current pulse shapes corresponding to these three isotope pairs, it can be observed that there is difficulty in discriminating between isotopes of similar energy, noting also that the most complicated case to solve is ^36,40^ Ar due to the higher overlap of their pulses throughout the graph. In this work, new methods will be developed to discriminate between these pairs of isotopes trying to achieve an algorithm based on computationally simple techniques that minimize the number of mathematical operations, avoiding divisions and non-linear operations. This is explained in the next section, Section 3.

## 3. Methodological Approach and Procedures

**Figure 2:**
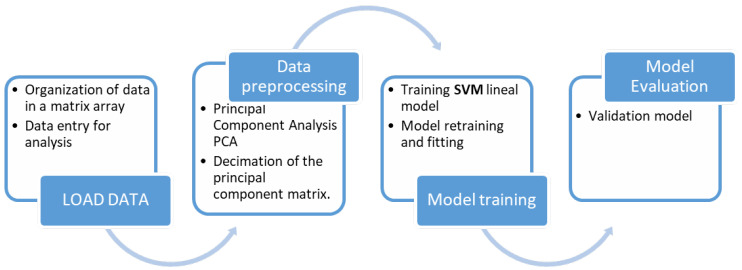
Workflow for obtaining the optimal SVM model.

This section describes the algorithm implemented to classify the detected isotopes. To demonstrate its performance, the dataset described in the previous section, Section 2, is used. Figure 2 describes the summary of the workflow to obtain the final isotope classifier.

The first stage of this workflow is to load the samples obtained for each isotope pair into memory, creating the necessary data structure. The information stored in this structure corresponds to the label, the name of the isotope with which the data are associated, and the number of protons (Z) and neutrons (N) that compose it. Once this step is completed, the second stage, denoted in Figure 2 as “Data preprocessing”, is performed. During this stage, the PCA technique is applied to reduce the number of features to be processed. This process is described in Section 3.1. After the reduction of the sample space, an SVM-based classifier is trained (Section 3.2). Finally, after the training phase, the generated model is applied to a new dataset to validate its estimation. All the coding works were implemented in Matlab, using the Statistics and Machine Learning Toolbox [33,34].

### 3.1. Data Pre-Processing: Principal Component Analysis

Principal component analysis is a statistical-algebraic technique that allows the dimensionality of a dataset to be reduced while preserving the maximum amount of information. This is achieved by linearly converting the data into a new coordinate system in which a significant portion of the data’s variation can be explained using fewer dimensions than the original dataset. This enables a reduction in the memory footprint of a dataset and significantly simplifies the classification algorithm without compromising precision [35]. For this work, the available dataset consists of the following feature vectors:

- ^80^Kr,1100 observations with 300 features
- ^84^Kr, 1100 observations with 300 features
- ^36^Ar, 2000 observations with 200 features
- ^40^Ar, 2000 observations with 200 features
- ^12^C, 2000 observations with 100 features
- ^13^C, 2000 observations with 100 features

Each observation corresponds to a sample vector with 300, 200, and 100 features for ^80,84^Kr,^36,40^Ar, and ^12,13^C isotopes, respectively. Note that each of the features corresponds to a sample of the electric current acquired by the 8-bit ACQIRIS digitizer, operating with a sample period of 1 ns. These vectors are arranged in a matrix for each isotope pair. The digitizer provides the samples with an interval of 1 ns. Thus, each observation has a duration of 300 ns, 200 ns, and 100 ns for Kr, Ar, and C isotopes, respectively. The digitizer output for each isotope pair is plotted in Figure 1. Note that the X-axis represents the time instant of the acquired sample and it can also be interpreted as the number of the sample, without loss of generality.

**Figure 3:**
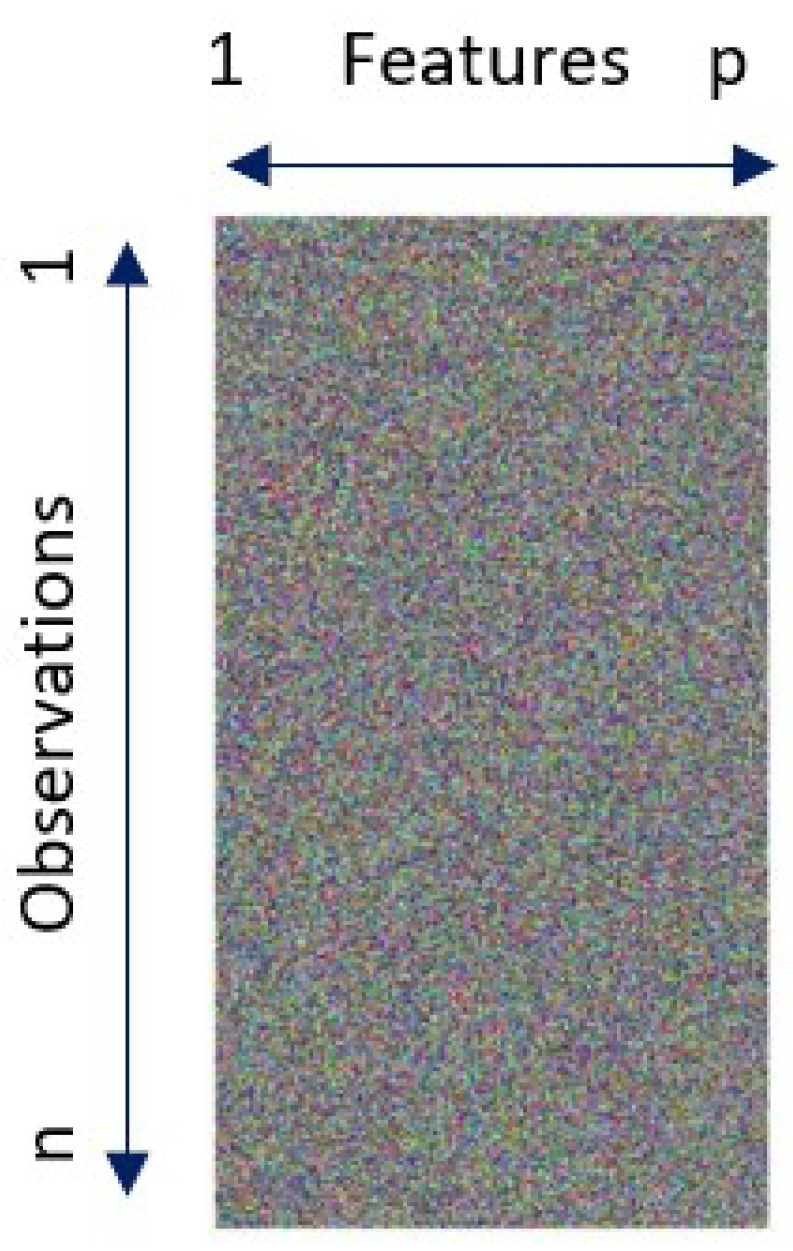
Visual example of a raw feature matrix.

Naturally, the aim of applying PCA is to reduce the amount of features to process in the classification techniques without compromising the accuracy and, hence, improving its computational efficiency. This reduction of features to be processed is beneficial since these techniques, see PCA, act as an enabling technology for the implementation of the edge-computing paradigm in the field of nuclear physics research. This new approach opens up the potential for processing and analyzing data in proximity to the silicon detector, thereby decreasing the amount of valuable data that need to be transmitted in an experiment. Furthermore, in the specific case of PCA, the way to generate the most relevant features of a dataset allows the resulting data to be used as a visualization tool, thus improving the understanding of the obtained data. Figure 3 shows a typical visual example that represents a dataset that could be the raw feature matrix corresponding to a certain isotope. Note that in this example, the matrix is composed of *n* observations and each observation has *p* features. Therefore, the sample space presents *p* dimensions.

**Figure 4:**
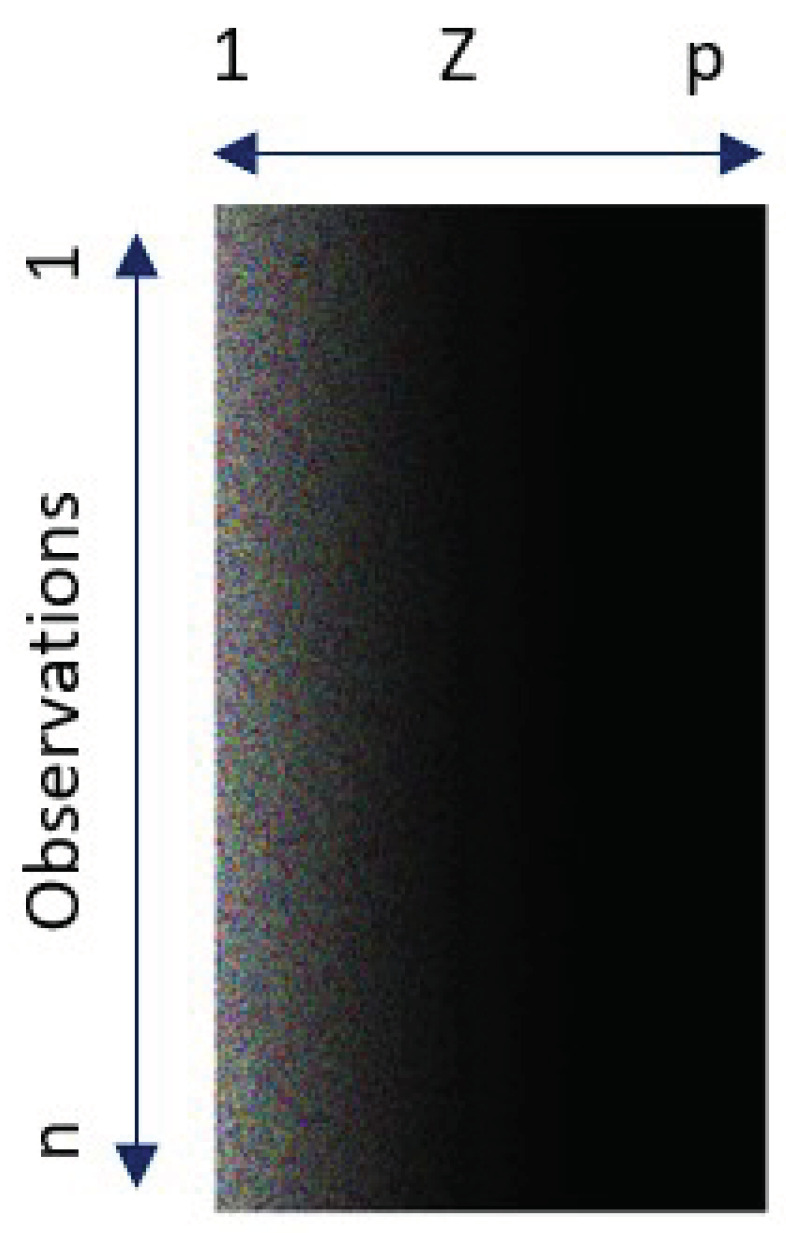
Visual example of a principal component matrix.

After applying PCA, the dimensionality of the dataset is reduced, generating a subset $Z=\{z_{1},z_{2},\ldots ,z_{j},\ldots ,z_{k}\}$, where *k* is a number much smaller than the original features *p*, k≪p. Figure 4 depicts the condensation of the information provided by multiple variables through PCA into just a few adjacent components.

Each principal component $z_{j}$ is obtained by a linear combination of the original variables. They can be understood as new features obtained by combining the original features in a certain way. The first principal component of a group of variables ($x_{1}$, $x_{2}$, …, $x_{p}$) is the normalized linear combination of these features that has the highest variance (1).

(1) $$z_{1}=\phi_{1,1}\;\cdot \;x_{1}+\phi_{2,1}\;\cdot \;x_{2}+\ldots +\phi_{p,1}\;\cdot \;x_{p}$$

The coefficients $\phi_{1,1},\ldots ,\phi_{1,p}$ are known as “loadings” and define each principal component, $z_{j}$. The loadings are understood as the weight that each feature has in each principal component, and it tells us what kind of information each component collects. These coefficients correspond to the eigenvector and eigenvalue of the covariance matrix.

**Figure 5:**
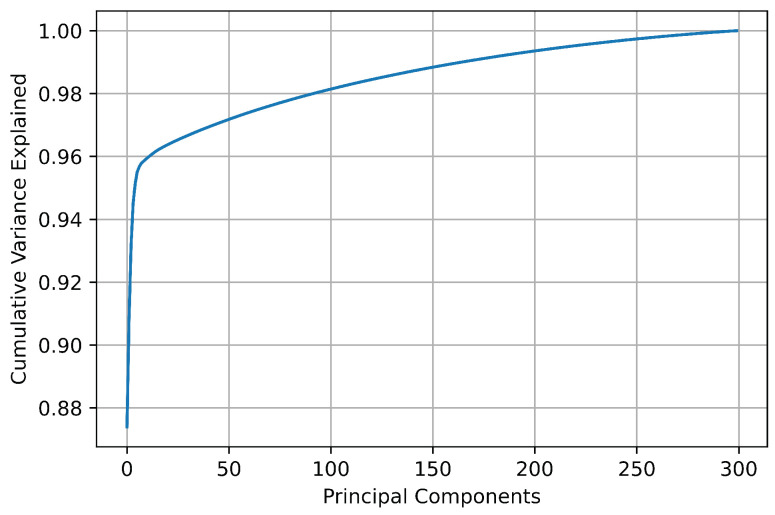
Cumulative explained variance of principal components.

For the dataset used in this work, a matrix of principal components of the same dimensions as the original dataset is obtained. However, because the purpose of PCA is to reduce the amount of data and retain as much information as possible, a minimum number of principal components must be found that are sufficient to preserve and explain the original features. There is no standardized solution or method to select the optimal number of principal components. Nevertheless, a widely accepted criterion is to evaluate the proportion of cumulative explained variance and select the minimum number of components beyond which the increase in variance is no longer significant. Figure 5 shows the cumulative explained variance of the principal components of the dataset for ^80,84^Kr. As can be observed, the greatest variation in the cumulative explained variance occurs in the first 20 principal components, where this parameter varies from 0.873959 for only one PC to 0.963423 for the first 20 PCs. From this number of principal components, the increase in the cumulative explained variance is not significant compared to the amount of data that must be processed.

### 3.2. Classifier: SVM

Support vector machines (SVMs) are a popular linear classifier based on supervised machine learning models [36,37], i.e., the sample dataset must be labeled to be used. Its effectiveness in classification, numerical prediction, and pattern recognition tasks has been exploited in this work to train an efficient model capable of identifying isotopes of similar energies. The aim of SVMs is to find a line or a hyperplane in dimensions greater than 2 between different classes of data such that the distance on either side of that line or hyperplane to the nearest data point is maximized. For this research, linear and cubic kernels are used.

In relation to the inputs of the model to be trained, these correspond to the characteristics generated by the PCA algorithm. Specifically, a series of principal components is evaluated that varies between 1 and 10. For each of these PCs, a different classifier model is trained. It is important to note that preprocessing the data with PCA not only contributes to reducing the number of characteristics of the observations, but also helps to maximize their variance; increasing the separation of some characteristics from others. A simple way to interpret this effect is from a geometrical point of view. The new features composed of the principal components occupy different positions in space and with greater separation between them. This allows for simplifying the line or hyperplane estimated by the SVM and, therefore, facilitating its classification.

The output of the classifier corresponds to the label associated with each of the observations. Thus, for each pair of isotopes, the classifier properly categorizes each one into its respective class and, once classified, assigns the corresponding number of neutrons and protons. These values are used in Section 4 to compare these algorithms with other classification techniques.

To train each model, the 80–20% rule was applied, i.e., 80% of the dataset was used for the training while the remaining 20% was dedicated to evaluate the accuracy of the model generated. Note that the accuracy is defined as the true positive predictions that are correct over the total number of cases. Furthermore, to obtain an accurate and robust classifier, a *k-fold* cross-validation strategy was applied to the training dataset. The main purpose of this method is to divide the dataset into multiple subsets or “folds”, allowing for training and testing the model multiple times. Specifically, the training dataset was subdivided into 5 sections so that during each training iteration composed of 5 iterations, 4 of these sections were used to train the model and the remaining one was used to evaluate it. Note that *k-fold-based* training was chosen since the amount of available data was limited, and, in these cases, this technique contributes to reduce the risk of overfitting, offering a more reliable estimate of the model’s performance. Finally, 40 iterations were run to obtain the final model. This procedure was applied for each of the chosen methods: linear and cubic.

**Figure 6:**
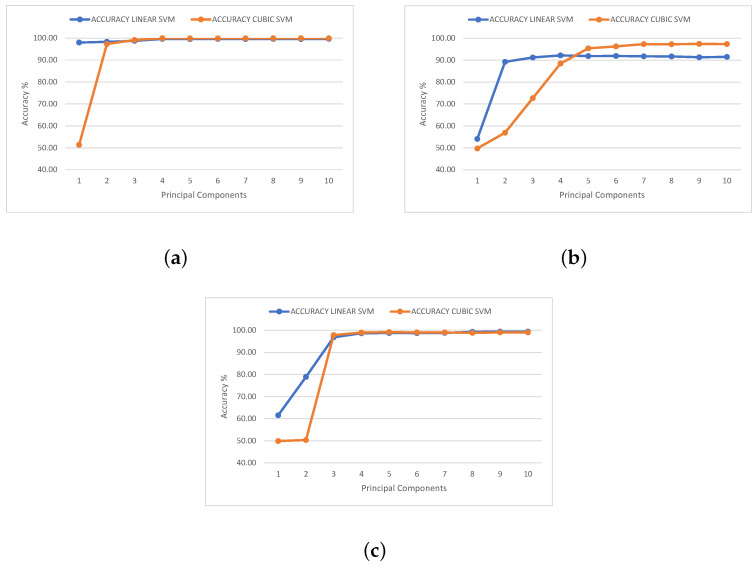
Accuracy for linear and cubic SVM algorithms for (**a**) ^12,13^C, (**b**) ^36,40^Ar, and (**c**) ^80,84^Kr.

To determine the optimal number of principal components, three metrics were considered for their evaluation: accuracy, merit factor, and the performance previously achieved by the neural network that we aim to surpass. In the case of the first metric, the precision of the SVM classifier, the estimation of the number of principal components to be used was performed according to two criteria. First, the success–error rate obtained after evaluating the confusion matrix must be higher than 90%, a value similar to that of other scientific publications [27,28]. The second defined criterion is that an increase in the number of principal components should represent a negligible percentage improvement in the classification. For this purpose, a percentage of 1% was established as the improvement threshold. Below this threshold, the computational resources required to implement the PCA algorithm and the SVM classifier increase significantly, leading to an increase in the complexity and size of a possible hardware implementation. Figure 6 presents the accuracy achieved by the model as a function of the different principal components used. The data represented in Figure 6 are summarized in Table 1 and Table 2.

The second metric is the merit factor. This metric describes the ability of the proposed SVM classifiers to discriminate between isotopes with similar energy, which are the most challenging classification cases in this type of study. In order to quantify the classification efficiency of our trained SVM models, measurements were conducted by estimating the degree of overlap between neighboring clusters. A widely used merit factor (FOM) *M* for gamma–neutron discrimination is presented in [22,38]. Equation (2) represents the generalized two-dimensional form of the merit factor *M*.

(2) $$M=\frac{\|\vec{\mu_{1}}-\vec{\mu_{2}}\|}{(\sigma_{1}+\sigma_{2})\;\cdot \;2.35}$$

where $\mu_{1,2}$ and $\sigma_{1,2}$ represent the corresponding two-dimensional centers and one-dimensional standard deviations of the classes, respectively [28].

**Figure 7:**
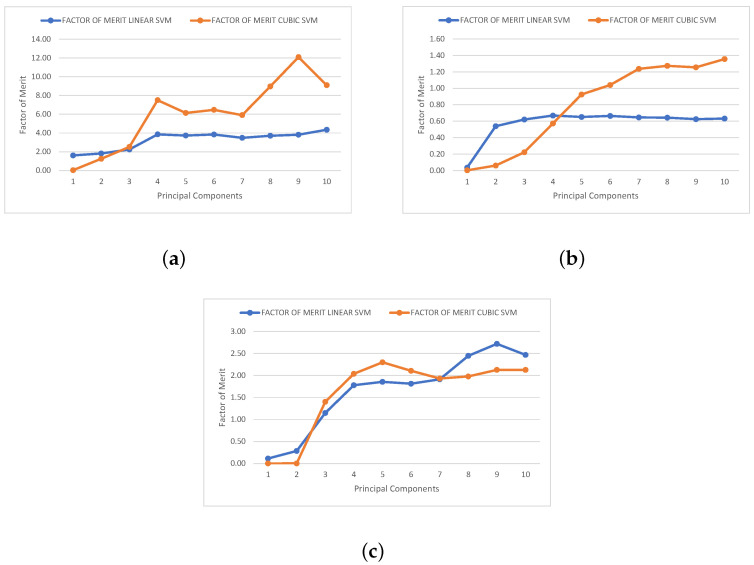
Factor of merit for linear and cubic SVM algorithms for (**a**) ^12,13^C, (**b**) ^36,40^Ar, and (**c**) ^80,84^Kr.

The merit factor is interpreted as follows: values of M>0.75 can be associated with a good rejection rate, and when M>1, almost all events are completely separated. To ensure acceptable discrimination between a pair of isotopes, the FOM must exceed 0.75; with the linear SVM, this value is adequately surpassed, except for the case of ^36,40^Ar. However, with the cubic SVM model, this threshold is exceeded for all three pairs of isotopes, ensuring proper discrimination and, therefore, an acceptable rejection rate. The merit factor data obtained for different numbers of principal components are displayed in Figure 7, and Table 3 and Table 4 represent these same results.

Finally, the third criterion for principal component selection is predicated on surpassing the previously established outcomes of the neural network referenced in this study. In other words, it is imperative that the merit factor value not only exceeds the minimum required by definition but also outperforms the classification capacity level of the reference neural network.

**Figure 8:**
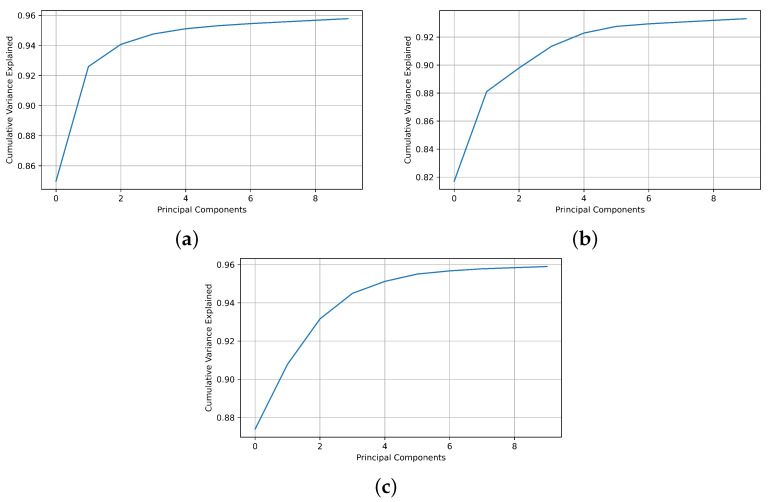
Cumulative explained variance for (**a**) ^12,13^C, (**b**) ^36,40^Ar, and (**c**) ^80,84^Kr.

Based on the results collected under the aforementioned three criteria, it can be observed that in the case of the isotope pairs ^12,13^ C and ^80,84^ Kr, the requisite number of principal components is four to achieve the required accuracy of the SVM classifiers and surpass the neural network in the cubic case. However, for ^36,40^ Ar, it is necessary to increase the number of principal components to six in order to attain an accuracy exceeding 92%. Hence, these numbers of principal components are employed to generate the final models, thereby conserving computational resources through classifier model simplification. Furthermore, these results were substantiated by cumulative explained variance, as depicted in Figure 8 for each isotope pair.

## 4. Identification between Pairs of Isotopes with Similar Energy

Once the optimal number of principal components required for the SVM classifier is determined, as detailed in the preceding section, the results of the classifier configured are presented in this section. Table 5 also contains evaluation metrics such as the prediction precision for each isotope; this metric is useful and reliable in cases where the dataset is symmetric between classes, and post PCA, our dataset is completely symmetric. This metric provides information about how often the true positive predictions are correct. It also contains the value of the merit factor calculated with Equation (2).

**Figure 9:**
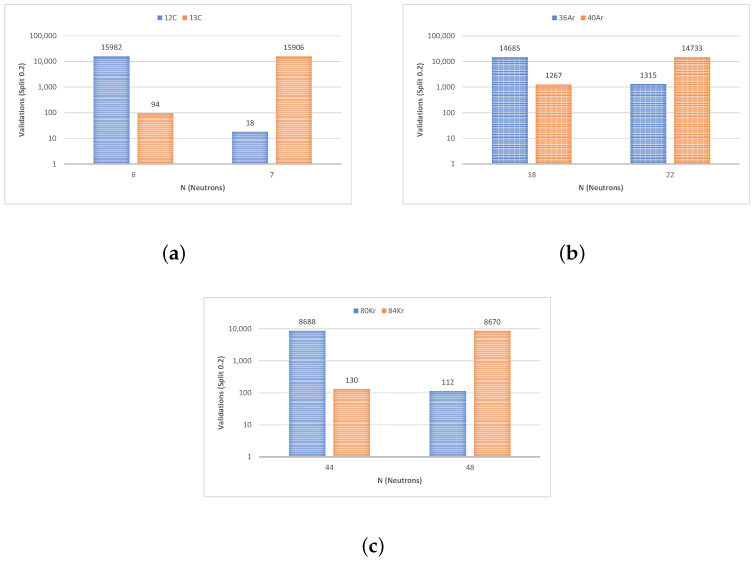
Success–error comparison on a logarithmic scale (from the confusion matrix) for linear SVM (**a**) ^12,13^C, (**b**) ^36,40^Ar, and (**c**) ^80,84^Kr.

**Figure 10:**
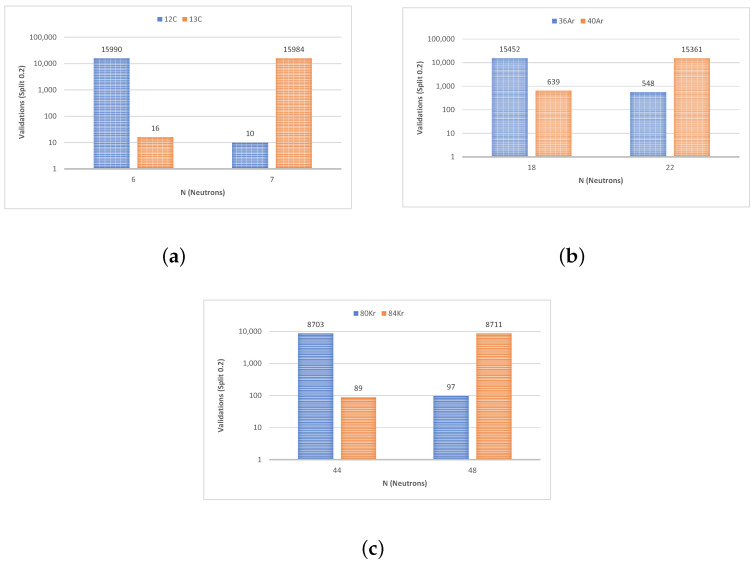
Success–error comparison on a logarithmic scale (from the confusion matrix) for cubic SVM (**a**) ^12,13^C, (**b**) ^36,40^Ar, and (**c**) ^80,84^Kr.

To describe the performance of our classification model, a validation tool known as a confusion matrix was used. Figure 9 and Figure 10 represent the values of each confusion matrix for each isotope pair to better understand the number of successes with respect to the total. Note that both figures were plotted on a logarithmic scale to improve their readability.

In order to validate the obtained results, a comparison is performed with the other methodologies previously documented in the scientific literature. Table 6 presents a comparison of the merit factors obtained through each method, including our own proposal.

From Table 6, it can be seen that the proposed classifiers achieve better classification results than the previously published methods. In addition, due to the reduction of the dataset due to the application of PCA, the required computational resources are optimized, as described in the next section.

## 5. Computational Cost

The relative efficiency of the algorithms is determined by comparing their computational complexities. In this section, the computational cost is evaluated considering the number of mathematical operations—number of additions and multiplications required—that each of the proposed algorithms (PCA + linear SVM, PCA + cubic SVM) require for their execution. Additionally, the theoretical resources occupied by a model based on neural networks [28] are also estimated in order to perform a proper comparison. Note that this methodology has been used previously in the literature [39].

### 5.1. Estimation

In order to determine the number of resources required for each method, the cost function collected in Table 7 was used.

**Figure 11:**
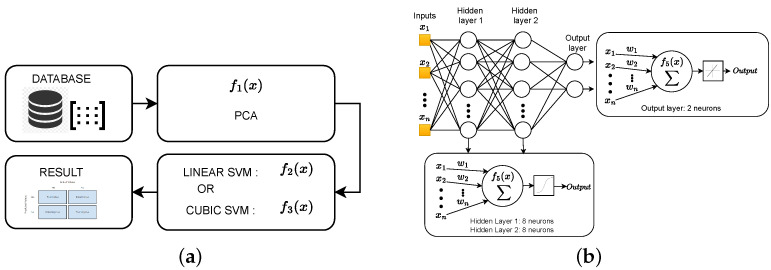
Schematic representation of the techniques (**a**) SVM and (**b**) ANN.

First, the estimation of the PCA algorithm was performed considering the function $f_{1}\left(x\right)$. This function represents the set of operations to be carried out to obtain the linear combination of each principal component, where $x_{i}$ represents the corresponding feature and $\phi_{i}$ denotes the loading factor associated with the feature $x_{i}$. Thus, the computation of a single principal component composed of *p* features requires Np multiplications and p−1 additions. Second, the resources required by the SVM algorithms were estimated using the functions $f_{2}\left(z\right)$ and $f_{3}\left(z\right)$. In both functions, *N* reflects the total number of principal components used to generate the model and $w_{i}$ is the weight associated with the PC. In the linear case, the term *b* is a constant value that represents the model bias. On the contrary, in the cubic SVM classifier, the terms $\alpha_{i}$ and $y_{i}$ correspond to the duality parameters and the observed response values, respectively. Regarding class encoding, $y_{i}=1$ denotes a positive feature, while $y_{i}=-1$ corresponds to a negative feature. In other words, y∈$\left\{1,-1\right\}$. Figure 11a depicts the data flow and the operations to be performed for each of the developed SVM-based classifiers.

Finally, to assess the computational neural network presented in [28], function $f_{4}\left(x\right)$ is used. In this function, *N* represents the total number of features, $w_{i}$ denotes the weights associated with neurons, $x_{j}$ corresponds to the input of the neural network, and *f* refers to an activation function [27,28]. The term *b* again denotes the bias of the model. Note that the neural network architecture comprises *n* inputs corresponding to the isotope-specific features, two hidden layers (each layer consists of eight neurons), and an output layer with two neurons. During the computation process, each neuron computes a weighted sum, where the input values are multiplied by their respective weights, and a bias term is added. Subsequently, the result of this weighted sum is subjected to an activation function. Two activation functions were used: the hyperbolic tangent sigmoid function and the “purelin” function, which determine the activation or excitation of the neuron, as depicted in Figure 11b.

Following the application of equation $f_{4}\left(x\right)$, Table 8 collects the number of operations carried out by the neural network for each pair of isotopes. This comprehensive information offers a quantitative assessment of the computational cost associated with this process, enabling a deeper understanding of the computational workload imposed by the neural network and its impact on the overall efficiency of the isotope classification process.

### 5.2. Analysis of Results

In this subsection is presented the number of additions and multiplications that are the operations performed by the PCA + linear SVM, PCA + cubic SVM, and neural network algorithms for each isotope pair. It can be seen from Table 9 that a significant amount of computational resources is required, regardless of the algorithm used.

For the ^12,13^C isotope pair, the neural network performs 3.02 and 4.85 times more operations, encompassing both additions and multiplications, compared to the PCA + cubic SVM; furthermore, it executes 3.05 and 4.35 times more operations than the PCA + linear SVM, respectively.

Concerning the ^36,40^Ar isotope pair, the neural network carries out 1.70 and 2.73 times more operations, involving additions and multiplications, in comparison to the PCA + cubic SVM; additionally, it conducts 1.71 and 2.78 times more operations than the PCA + linear SVM, respectively.

Finally, for the ^80,84^Kr isotope pair, the neural network performs 5.46 times more additions and 2.07 times more operations, covering both additions and multiplications, compared to the PCA + cubic SVM; moreover, it executes 5.48 and 2.09 times more operations than the PCA + linear SVM, respectively.

## 6. Conclusions

Data preprocessing through PCA has proven to be an effective strategy for reducing the information load without compromising the results. The selection of four principal components for the isotope pairs ^12,13^C and ^80,84^Kr and six principal components for ^36,40^Ar, based on cumulative explained variance analysis, has allowed the condensation of most of the features into these components, which is sufficient to achieve the required accuracy. Note that, in spite of the similarity between the energy levels of the ^36,40^Ar isotope pair, which corresponds to one of the most challenging isotopes to discriminate, our cubic SVM model has demonstrated significantly more efficient classification compared to the linear SVM. The proposed approach in this work for classifying isotopes with similar energy levels has proven to provide high precision in comparison to the other methodologies present in the literature, such as ANNs. Furthermore, the validation metrics and merit factor used have met expectations. Regarding the computational cost analysis, our results indicate that SVM algorithms require fewer computational resources in terms of ’operations’ than ANNs, highlighting the efficiency of this technique in isotope classification applications. The significant reduction in computational cost opens up the possibility of implementing these models for isotope classification in the context of edge computing.

## Figures and Tables

**Table 1:** Accuracy values obtained for linear SVM.

Isotope	Number of Principal Components									
	1	2	3	4	5	6	7	8	9	10
^12,13^C	98.00	98.30	98.73	99.65	99.58	99.66	99.60	99.61	99.57	99.67
^36,40^Ar	54.07	89.24	91.21	92.18	91.86	91.93	91.75	91.69	91.32	91.48
^80,84^Kr	61.54	78.88	96.84	98.62	98.73	98.68	98.80	99.25	99.39	99.35

**Table 2:** Accuracy values obtained for cubic SVM.

Isotope	Number of Principal Components									
	1	2	3	4	5	6	7	8	9	10
^12,13^C	51.32	97.29	99.14	99.92	99.88	99.88	99.87	99.94	99.97	99.94
^36,40^Ar	49.71	56.93	72.66	88.43	95.39	96.29	97.33	97.28	97.42	97.35
^80,84^Kr	49.90	50.34	97.81	98.94	99.16	99.01	99.01	98.81	99.03	99.03

**Table 3:** Factor of merit obtained for linear SVM.

Isotope	Number of Principal Components									
	1	2	3	4	5	6	7	8	9	10
^12,13^C	1.61	1.81	2.24	3.84	3.73	3.84	3.48	3.70	3.81	4.35
^36,40^Ar	0.04	0.54	0.62	0.66	0.65	0.66	0.65	0.64	0.62	0.63
^80,84^Kr	0.11	0.29	1.15	1.78	1.85	1.81	1.91	2.45	2.72	2.47

**Table 4:** Factor of merit obtained for cubic SVM.

Isotope	Number of Principal Components									
	1	2	3	4	5	6	7	8	9	10
^12,13^C	0.03	1.24	2.51	7.50	6.14	6.47	5.90	8.96	12.09	9.09
^36,40^Ar	0.00	0.06	0.22	0.57	0.92	1.04	1.24	1.27	1.26	1.36
^80,84^Kr	0.00	0.00	1.40	2.04	2.30	2.11	1.93	1.98	2.13	2.13

**Table 5:** Evaluation metrics of the SVM classifier algorithms.

Isotope	Linear SVM		Cubic SVM	
	Evaluation Metrics [%]	M	Evaluation Metrics [%]	M
^12,13^C	99.88–99.65	3.84	99.94–99.92	7.50
^36,40^Ar	91.78–91.93	0.66	96.57–96.29	1.04
^80,84^Kr	98.73–98.62	1.78	98.90–98.94	2.04

**Table 6:** Factor of merit among different methods.

Methods	Units	Reference	M per Isotope Pair		
			^12,13^C	^36,40^Ar	^80,84^Kr
Amplitude	[mA]	[22]	1.42	0.81	0.54
Risetime	[ns]	[22]	0.62	0.36	0.26
Decay time	[ns]	[22]	0.81	0.48	0.007
Slope	[mA/ns]	[22]	1.35	0.73	0.11
m${}^{2}$	[ns]	[22]	0.91	0.64	≈0
f[i] current signal	-	[22]	1.15	0.84	0.50
data[i] current signal	-	[22]	1.53	0.96	1.04
Standard ANN	-	[28]	1.71	0.76	0.98
Differential ANN	-	[28]	4.48	0.90	2.95
Linear SVM	-	This work	3.84	0.66	1.78
Cubic SVM	-	This work	7.50	1.04	2.04

**Table 7:** Algorithm Cost Functions.

Method	Cost Function
PCA	$f_{1}\left(x\right)=\sum_{i=1}^{p}\phi_{i}x_{i}$
Linear SVM	$f_{2}\left(z\right)=b+\sum_{i=1}^{N}w_{i}\;\cdot \;z_{i}$
Cubic SVM	$f_{3}\left(z\right)=\sum_{i=1}^{N}\alpha_{i}y_{i}{\left(1+w_{i}\;\cdot \;z_{i}\right)}^{3}$
ANN ([28])	$f_{4}\left(x\right)=f\left(\sum_{j=1}^{N}w_{j}x_{j}+b\right)$

**Table 8:** Computational cost of the neural network.

Computational Cost (ANN)	^12,13^C		^36,40^Ar		^80,84^Kr	
	Sums	Products	Sums	Products	Sums	Products
Hidden layer 1	1600	824	3200	1624	4800	2424
Hidden layer 2	128	88	128	88	128	88
Output layer	30	16	30	16	30	16

**Table 9:** Computational cost of the neural network and proposed methods for comparison.

Computational Cost	Functions	^12,13^C (4-PCA)		^36,40^Ar (6-PCA)		^80,84^Kr (4-PCA)	
		Sums	Products	Sums	Products	Sums	Products
PCA + linear SVM	$f_{1}\left(x\right)+f_{2}\left(x\right)$	304	404	1006	1206	904	1204
PCA + cubic SVM	$f_{1}\left(x\right)+f_{3}\left(x\right)$	307	420	1011	1230	907	1220
ANN	$f_{4}\left(x\right)$	928	1758	1728	3358	4958	2528

## Data Availability

Data available on request.

## Abbreviations

The following abbreviations are used in this manuscript:

AI	Artificial intelligence
ANN	Artificial neural network
DPSA	Digital pulse shape analysis
EURISOL	European isotope separation on-line
FAIR	Facility for antiproton and ion research
FAZIA	Four-pi A and Z identification array
FPGA	Field-programmable gate array
ML	Machine learning
N	Neutrons
NTD	Neutron transmutation doped
PC	Principal component
PCA	Principal component analysis
PSA	Pulse shape analysis
RIBF	Radioactive ion beam facility
SPES	Selective production of exotic species
SVM	Support vector machine
ToF	Time-of-flight
Z	Protons
